# Watersiporidae (Bryozoa) in Iberian waters: an update on alien and native species

**DOI:** 10.1007/s12526-019-01003-4

**Published:** 2019-11-14

**Authors:** Oscar Reverter-Gil, Javier Souto

**Affiliations:** 1 Museo de Historia Natural da Universidade de Santiago de Compostela, Parque Vista Alegre s/n, 15705 Santiago de Compostela, Spain; 2 Institut für Paläontologie, Geozentrum, Universität Wien, Vienna, Austria; 3 Facultade de Bioloxía, Departamento de Zooloxía, Xenética e Antropoloxía Física, Universidade de Santiago de Compostela, Santiago de Compostela, Spain

**Keywords:** Non-indigenous species, *Watersipora*, New genus, NE Atlantic, Western Mediterranean, Strait of Gibraltar, Spain, Portugal

## Abstract

Species of the genus *Watersipora* comprise an important invasive fouling group but are difficult to identify up to species level. This problem, in conjunction with the recent re-description of several member species, requires the revision of previous records and newly collected material in order to more precisely determine their actual presence and distribution. Here, we revise the identity and distribution of alien and native species of Watersiporidae in Iberian waters based on newly collected material, historical collections, and bibliographic data. Four species of *Watersipora* are now known from here. *Watersipora cucullata* is the only native species, present in the Spanish Mediterranean. *Watersipora subatra* seems to have been introduced relatively recently in Iberian and European Atlantic waters and has been expanding to other Atlantic localities. *Watersipora arcuata* was collected for the first time in Europe at the SW Spanish Atlantic coast in 1990 and recently in Mediterranean marinas. *Watersipora souleorum* is known in Iberian waters from two localities in the Gulf of Cadiz and in Gibraltar. With the recent redescription of the genus *Watersipora*, *W. complanata* is no longer a member. A new watersiporid genus, *Terwasipora* gen. nov., is described for this species. In Iberian waters, *T. complanata* comb. nov. is considered a native species, frequent and abundant in shallow waters along the Atlantic coast.

## Introduction

The bryozoan family Watersiporidae currently includes three genera (Bock and Gordon 2018a). Two of them are monospecific, with the species *Uscia mexicana* Banta, 1969a, from the Gulf of Mexico and *Veleroa veleronis* Osburn, 1952, from the eastern Pacific. The third genus, *Watersipora* Neviani, 1896, is the largest one, both by number of species (13 recent species according to Bock and Gordon 2018b) as well as by geographical distribution, as it is found around all oceans, mainly in tropical and subtropical areas. It is also the only member of the family that has been reported in European waters.

The morphology of the genus *Watersipora* is simple in comparison to other genera of cheilostome bryozoans because certain diagnostic structures such as avicularia, spines, or ooecia, which in other groups of bryozoans are very useful for distinguishing between species, are lacking. Several species are therefore difficult to differentiate without using SEM images, morphometric characters, or examining operculum details (Vieira et al. 2014). Moreover, some species of *Watersipora* are important invasive fouling organisms, reported in harbor areas all around the world (Gordon and Mawatari 1992; Mackie et al. 2012; Ulman et al. 2017). This wide dispersion, together with their simple morphology, lack of valid type material, or accurate descriptions of several species until recently, has produced widespread confusion (Ryland et al. 2009; Vieira et al. 2014). The same name has frequently been used by different authors to designate different species; vice versa, a single species has often received different names in different places by various authors.

Ryland et al. (2009) noted that *Watersipora subtorquata* (d’Orbigny, 1852) and *W. subovoidea* (d’Orbigny, 1852) were frequently confused due to the absence of a modern taxonomic account comparing both species. To solve this problem, they designated a neotype for *W. subovoidea* and established its conspecificity with *W. cucullata* (Busk, 1854), but no redescription of the type material of *W. subtorquata* was given. Soon after, Vieira et al. (2014) disagreed with some of the conclusions reached by Ryland et al. (2009). They redescribed *W. subtorquata* and *W. cucullata* based on original material and stated that most of the material assigned to *W. subovoidea* (a species that would no longer be valid) actually corresponds to *W. subtorquata*. Nonetheless, other specimens previously assigned to *W. subtorquata* belong to *W. subatra* (Ortmann, 1890), a species also redescribed by Vieira et al. (2014). In the present paper, we follow the conclusions reached by these authors.

*Watersipora subtorquata*, *W. subovoidea*, and *W. cucullata* have been reported from several localities in Iberian waters. Taking into account the continuous mistakes, however, confirmed identifications call for the revision of the original material of previous records or, in very rare cases, the examination of complete descriptions and figures. Finally, *Watersipora souleorum* Vieira, Spencer Jones & Taylor, 2014 has very recently been reported from an unknown Atlantic Spanish locality (among other localities around the world) (Vieira et al. 2014) and *Watersipora arcuata* Banta, 1969b from the Spanish Mediterranean coast (Ulman et al. 2017).

In the case of *Watersipora complanata* (Norman, 1864), also reported in Iberian waters, the problem is different. It is a relatively well-known species, but some aspects of its morphology remain unknown: the ancestrula and early astogeny, for example, are described below for the first time. This lack of knowledge has obscured the systematic relationships of this species, which were long uncertain. Although the species was placed in the genus *Watersipora* (see Hayward 1976) 40 years ago, Ryland et al. (2009) already indicated some doubts about its taxonomic placing. Therefore, in the present work, we revise previous records of *Watersipora* in Iberian waters, together with newly collected material.

## Material and methods

Samples were studied, which were collected during different campaigns and projects around the Iberian Peninsula, from the intertidal to 32 m depth. This material was sampled from natural and artificial substrates, and the specimens were conserved in alcohol. Samples from Santander, Asturias, and Galicia were collected by the authors on different dates by SCUBA and also in the intertidal. This material is now held at the Museo de Historia Natural da Universidade de Santiago de Compostela (MHNUSC-Bry) (Figs. 7 and 8). Samples from Portugal were collected in the frameworks of the campaigns EMEPC/M@rBis/Berlengas2012, EMEPC/M@rBis/Arrábida2014, and EMEPC/M@rBis 2015 carried out on the Berlengas Islands, Arrábida coast, and Lisbon coast, respectively, and organized by Estrutura de Missão para a Extensão da Plataforma Continental (EMEPC). During these campaigns, bryozoans were collected at 85 localities by SCUBA and by wading in the intertidal; Watersiporidae were collected in only 16 localities. In Portugal, samples collected during the biological monitoring program at the Ocean Revival underwater park (Algarve) were also studied; for details of the park and experiments, see Encarnaçao and Calado (2018) (Figs. 7 and 8).

In order to verify the identification of previous records, historical specimens held at different institutions were also reexamined: Museo de Historia Natural da USC (MHNUSC), Museo Nacional de Ciencias Naturales, Madrid (MNCN), and Muséum National d’Histoire Naturelle, Paris (MNHN), as well as several samples from the personal collection of C.M. López-Fé, now held at the MHNUSC. Details of the localities and specimens identified are presented in the sections for each species below.

Samples were sorted and examined in the lab using stereomicroscopes. Selected specimens were cleaned and dried for study by scanning electron microscopy (SEM). An FEI Inspect S50 SEM and Zeiss EVO LS15, from the University of Vienna and the University of Santiago de Compostela, respectively, were used to take photographs of uncoated material with a back-scattered electron detector in low-vacuum mode. Measurements were taken with the software ImageJ® on SEM photographs. Optical photos were taken with a Leica DFC 425 digital camera. Field photographs were made with a Nikon D90 digital camera. The material newly collected during the present work is stored in the bryozoan collection at the Museo de Historia Natural of the USC (MHNUSC-Bry).

## Results

### Systematics

Family Watersiporidae Vigneaux, 1949

Genus ***Watersipora*** Neviani, 1896

### *Watersipora cucullata* (Busk, 1854)

**Fig. 1:**
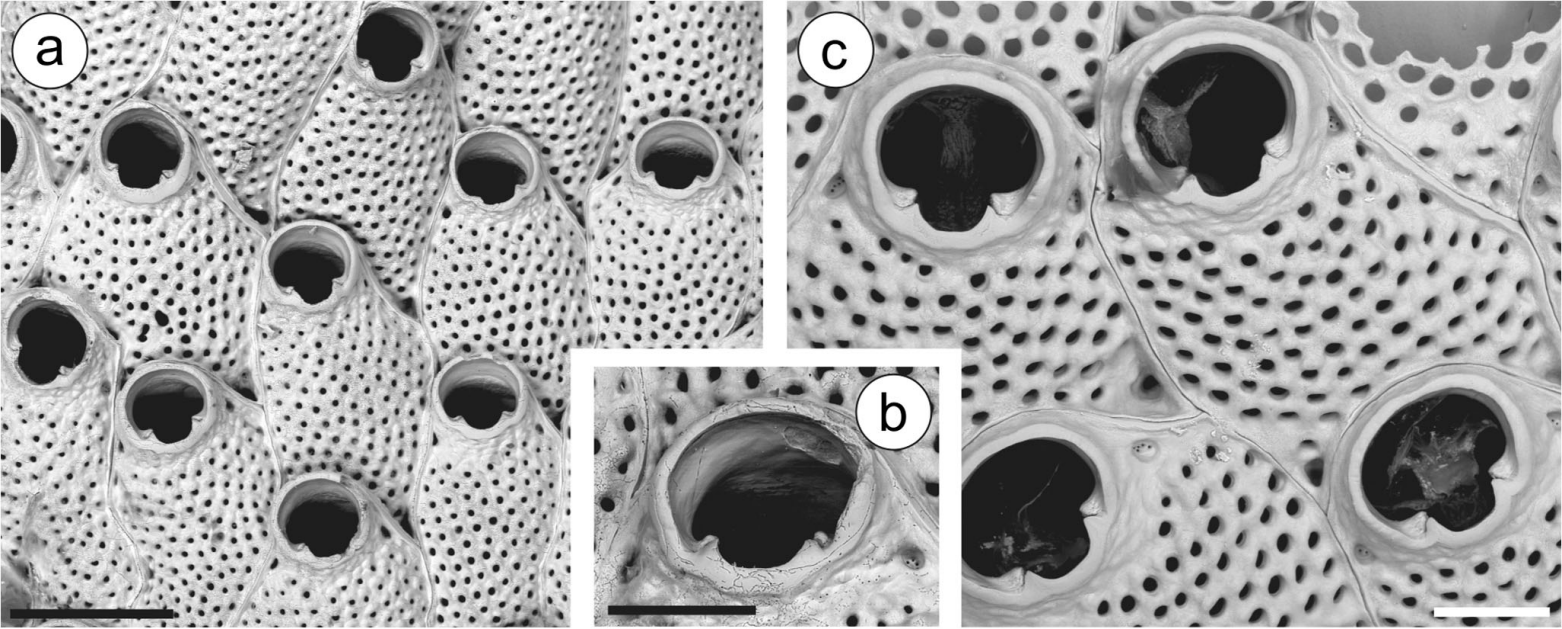
*Watersipora cucullata*. **a** Part of the colony (MNCN 25.03/3042: Islas Columbretes); **b** same, detail showing the intrazooidal septula; **c** detail of the zooidal orifices and intrazooidal septula (MNHN-IB-2008-11524: Castiglione, Algeria). Scale bars: **a** 0.5 mm; **b**, **c** 0.2 mm

(Figs. 1, 7) *Lepralia cucullata* Busk, 1854: 81, pl. 96, figs. 4 and 5.

Not *Watersipora cucullata*: Calvet 1931: 113 (part: Gibraltar) [=*Watersipora souleorum* Vieira et al., 2014].

*Watersipora subovoidea*: Gautier 1962: 183 (part: Castiglione, Algeria); Zabala 1986: 396, pl. 6A; Hayward and McKinney 2002: 63, fig. 29A-B; Templado et al. 2002: 203; Chimenz-Gusso et al. 2014: 308, fig. 173a-d.

Not *Watersipora subovoidea*: López de la Cuadra 1991: 197, pl. 2, fig. B, pl. 21, fig. H, pl. 22 fig. A [=*Watersipora souleorum* Vieira et al., 2014 and *Watersipora arcuata* Banta, 1969b].

Not *Watersipora subovoidea*: César-Aldariz et al. 1997: 215, figs. 6 and 7; Reverter-Gil and Fernández-Pulpeiro 2001: 121. [=*Watersipora subatra* (Ortmann, 1890)].

*Watersipora cucullata*: Vieira et al. 2014: 16, figs. 6–9, 25–34, 65.

### Material examined

**MediterraneanSpain**—MNCN 25.03/784, 1350: Isla Espardell (Isla de Ibiza), 38.79833°N 01.48250°E, 20–25 m depth (Fauna III); MNCN 25.03/2118: Columbrete Grande (Islas Columbretes), 39.90000°N 0.68500°E, 30 m depth (Fauna IV); MNCN 25.03/2986: Islas Columbretes, 285 B22, 39.85450°N 0.67617°E, 30 m depth (Fauna III); MNCN 25.03/2996, 3079: La Horadada (Islas Columbretes), 39.87283°N 0.66967°E, 21.5 m depth (Fauna IV); MNCN 25.03/3011, 3042: Islote Churruca (Islas Columbretes), 39.85450°N 0.67633°E, 42 m depth (Fauna IV) (Fig. 1a, b); MNCN 25.03/3062: Banco El Fidalgo (Islas Columbretes), 39.89733°N 0.667833°E, 3–40 m depth (Fauna IV); MNCN 25.03/3414, 4039: La Horadada (Islas Columbretes), 39.87283°N 0.66967°E, 24 m depth (Fauna IV). **Mediterranean Algeria**—MNHN-IB-2008-11524: Castiglione. Gautier Coll. (labeled as “*Watersipora*”) (Fig. 1c).

### Description

Colony encrusting, multiserial, blackish in color. Zooids subrectangular to hexagonal, 0.77 to 1.02 mm long by 0.39 to 0.69 mm wide, separated by slightly raised grooves. Frontal shield uniformly perforated by round pseudopores, but obscured by a thick, black epitheca; two intrazooidal septula proximolateral to the orifice, each with 3–5 small pores. Orifice large, slightly wider than long, 0.20 to 0.24 mm long by 0.23 to 0.27 mm wide, with a well-defined U-shaped sinus demarcated by triangular projections; orificial rim thickened around whole orifice; condyles upturned, conspicuous. Operculum with broad, parallel-sided dark central band and slightly thinner lateral area. Spines, avicularia and ooecia absent.

### Remarks

As already stated above, *W. cucullata* has been recently redescribed by Vieira et al. (2014) based on the original material of Busk (1854). In Iberian waters, Vieira et al. (2014) reported a sample of *W. cucullata* coming from the Balearic Islands, without further information about the locality (NHMUK 1965.8.14.10). Zabala (1986) reported *W. subovoidea* from the Catalan coast (Medes Islands); although the description does not allow drawing conclusions about the identity of the material, the photograph shows the zoecial orifice (Zabala 1986, pl. 6A) which undoubtedly corresponds to *W. cucullata*. Templado et al. (2002) reported *W. subovoidea* from the Columbretes Islands (Mediterranean Spain) but the reference material conserved at the MNCN (see material examined and Fig. 1a, b) actually belongs to *W. cucullata*. The records from the same archipelago made by d’Hondt (1979) and Saguar and Boronat (1987) as *W. subovoidea* probably belong to the same species, but no material has been preserved. In addition, two unpublished samples from Ibiza (Balearics, Mediterranean Spain: see material examined) also belong to *W. cucullata*.

There are several old records of *W. cucullata* from different Iberian Mediterranean localities: Gibraltar (Barroso 1917); Valencia and Denia (Barroso 1921); and Mallorca (Barroso 1923, Gautier 1957 and 1962 as *W. subovoidea*). The original material of these references has not been conserved, and in some cases the records are only nominal, while in others the descriptions and figures do not allow to verify the identifications; all of them must therefore be considered doubtful.

There are also two other Mediterranean records, namely *W. subovoidea* by Calvín Calvo (1986) from Isla Grosa (Murcia, Mediterranean) and by Zabala (1993) from Cabrera (Balearics), but the lack of reference material prevents verifying their identifications. Nonetheless, the record by Zabala (1993) could correspond to *W. cucullata*, a species already reported in that archipelago.

Finally, the record of *W. cucullata* from Gibraltar made by Calvet (1931) corresponds to *Watersipora souleorum* Vieira, Spencer Jones and Taylor, 2014 (see below). In any case, the existence of *W. cucullata* in Iberian Mediterranean waters is confirmed, which is not surprising since this species seems to be endemic in the Mediterranean.

### *Watersipora subatra* (Ortmann, 1890)

**Fig. 2:**
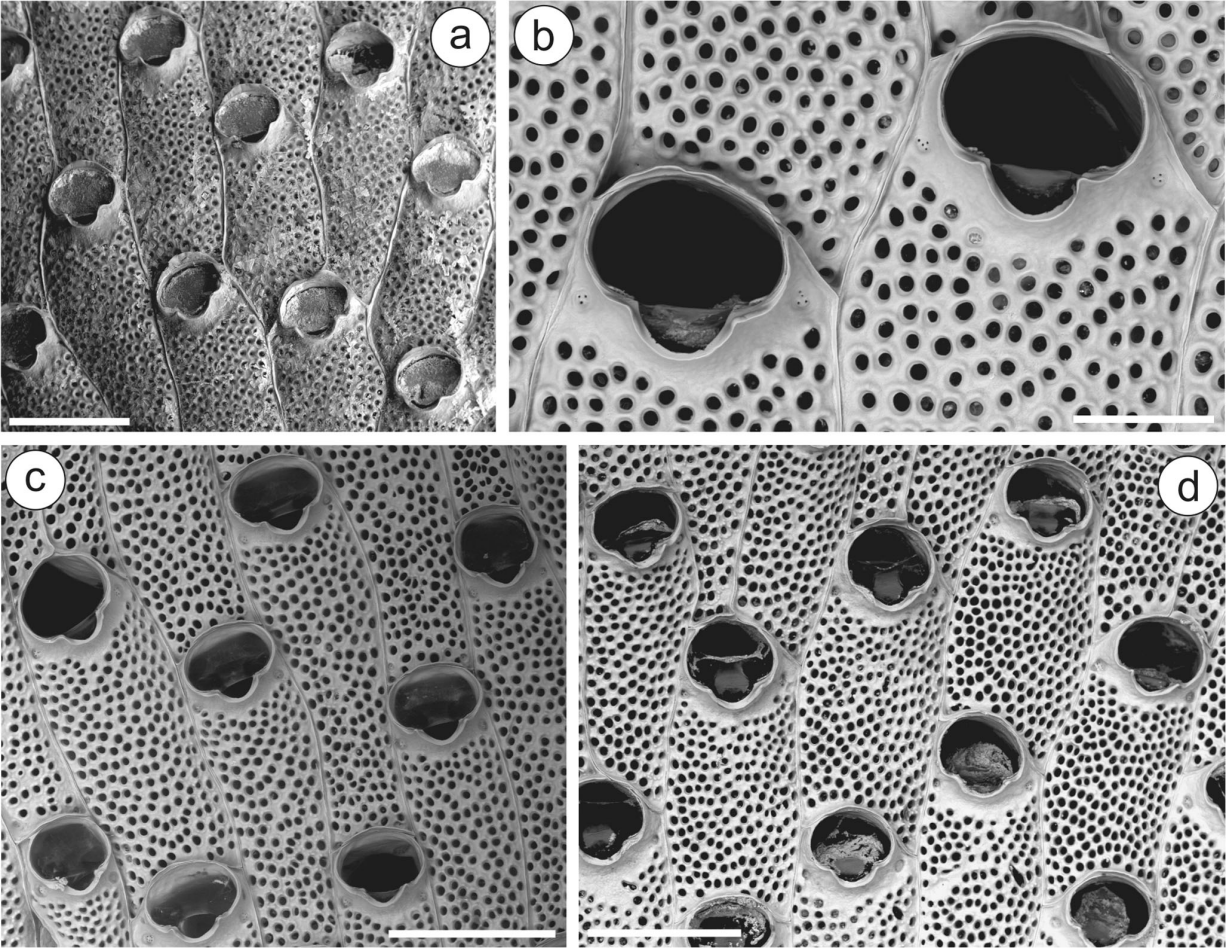
*Watersipora subatra*. **a** Uncleaned colony from Santander (MHNUSC-Bry 665); **b** detail of the zooidal orifices and intrazooidal septula from Ferrol (MHNUSC-Bry 674); **c** detail of a colony from Vigo (MHNUSC-Bry 673); **d** detail of a colony from the Algarve (MHNUSC-Bry 437). Scale bars: **a**, **c**, **d** 0.5 mm; **b** 0.2 mm

**Fig. 3:**
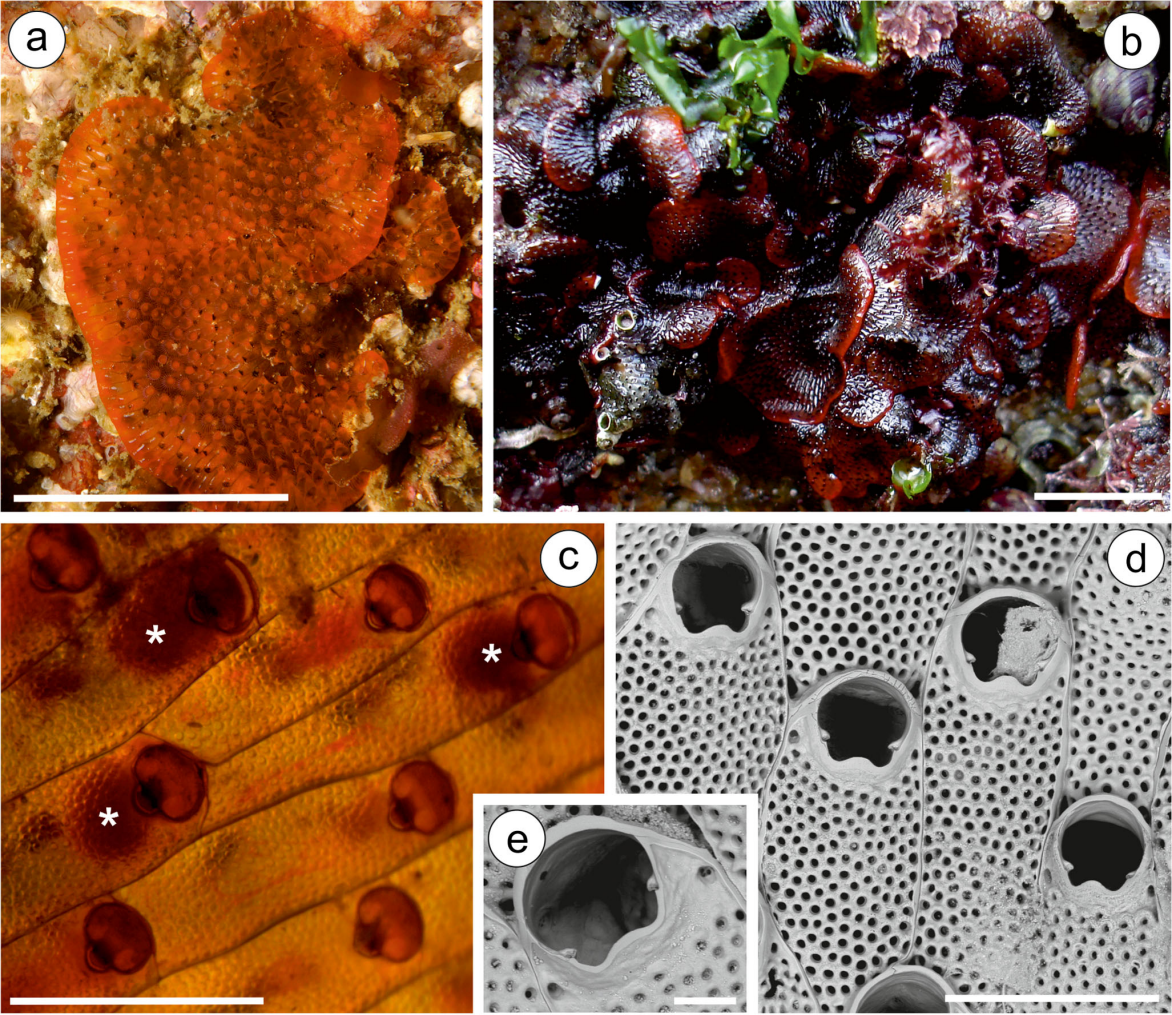
**a–c***Watersipora subatra*. **a** Young colony showing the tentacular crowns (Ferrol: San Carlos); **b** colony with lobes (Xove: Morás); **c** autozooids with embryos (asterisks) (Ferrol: A Graña); **d** autozooids of *Watersipora arcuata* (MHNUSC-Bry 471: Caños de Meca, Cádiz); **e** same, detail showing the intrazooidal septula. Scale bars: **a**, **b** 10 mm; **c** 1 mm; **d** 0.5 mm; **e** 0.1 mm

(Figs. 2, 3a-c, 7)*Schizoporella aterrima* var. *subatra* Ortmann, 1890: 49.

*Watersipora subovoidea*: César-Aldariz et al. 1997: 215, figs. 6 and 7; Reverter-Gil and Fernández-Pulpeiro 2001: 121.

Not *Watersipora subovoidea*: Gautier 1962: 183 (part: Castiglione, Algeria); Zabala 1986: 396, pl. 6A; Hayward and McKinney 2002: 63, fig. 29A-B; Templado et al. 2002: 203; Chimenz Gusso et al. 2014: 308, fig. 173a-d [=*Watersipora cucullata* (Busk, 1854)].

Not *Watersipora subovoidea*: López de la Cuadra 1991: 197, pl. 2, fig. B, pl. 21, fig. H, pl. 22 fig. A [=*Watersipora souleorum* Vieira et al., 2014 and *Watersipora arcuata* Banta, 1969b].

*Watersipora subtorquata*: Ryland et al. 2009: 55, figs. 3, 4A, C, E, F; Souto et al. 2014: 145 (part: Cascais and Portimão); Reverter-Gil et al. 2014: 26 (part: Cascais and Portimão).

Not *Watersipora subtorquata*: Souto et al. 2014: 145, fig. 6F (part: Faro); Reverter-Gil et al. 2014: 26 (part: Faro) [=*Watersipora souleorum* Vieira et al., 2014].

*Watersipora subatra*: Vieira et al. 2014: 166, figs. 39–53, 66, 69.

### Material examined

**N Spain**—MHNUSC-Bry 663: Santander, Rampa Gamazo, 43.46145°N 03.78930°W, intertidal, 05/01/2018; MHNUSC-Bry 664, 665: Santander, Puerto Chico, 43.46246°N 03.79366°W, on pontoons, buoys and ropes, 05/01/2018 (Fig. 2a); MHNUSC-Bry 671, 672: Asturias, Gijón, El Musel, 43.55170°N 05.68833°W, pontoons, 19/05/2019. **NWSpain**—MHNUSC-Bry 669: Cabo Prior: Ponzos, 43.55680°N 08.26402°W, on a plastic bottle washed upon the beach, 31/12/2017; MHNUSC-Bry 362, 363, 674: Ría de Ferrol: San Felipe, 43.46580°N 08.28040°W, intertidal, 30/01/2010 (Fig. 2b); MHNUSC-Bry 666, 667, 668: Ría de Ferrol: A Graña, 43.47917°N 08.25972°W, 02/01/2018; MHNUSC-Bry 361, 673: Ría de Vigo: Pta. Creixiña, Cangas, 42.25417°N 08.83333°W, 5 m depth, 15/10/2010 (Fig. 2c); MHNUSC-Bry 474: Ría de Vigo: Bouzas, 42.22825°N 08.74952°W, intertidal, 11/2017; MHNUSC-Bry 364: Cíes Islands, 42.23871°N 08.89948°W, 6 m depth, 22/08/2012. Other localities (material without reference number): Xove: Morás beach, 43.71833°N 07.47254°W, 27/09/2007 (Fig. 3b); Ría de Ferrol: San Carlos, 43.46094°N 08.29856°W, 21/08/2016 (Fig. 3a); Ría de Ferrol: Laxe, 43.46469°N 08.28422°W, 20/05/2017; Ría de Ferrol: Santa Lucía, 43.46056°N 08.24500°W; Ría de Ferrol: A Graña, 43.47917°N 08.25972°W, 04/10/2011 (Fig. 3c); Ría de Ferrol: D22, 43.46250°N 08.24361°W, 12 m depth; Ría de Ferrol: Punta Piteira, 43.46444°N 08.26194°W, 5–16 m depth, 05/2019. **Portugal**—MHNUSC-Bry 675: Marina of Cascais, 38.69288°N 09.41726°W, 30/05/2015; MHNUSC-Bry 676: Lisbon, Marina of Belén, 38.69446°N 09.20405°W, 01/06/2015, 07/04/2016; MHNUSC-Bry 677: Lisbon, Marina of Alcantara, 38.70145°N 09.16933°W, 21/03/2016; MHNUSC-Bry 678: Lisbon, Marina of Oeiras, 38.67617°N 09.31794°W, 15/04/2016; MHNUSC-Bry 679: Lisbon, Marina of Almada, 38.67250°N, 09.14197°W, 04/10/2014; MHNUSC-Bry 680: Sesimbra harbor, 38.43663°N 09.11385°W, on *Mytilus* and buoys, 29/09/2014; MHNUSC-Bry 670: Algarve: Portimão, 37.091683°N 08.582733°W, Ocean Revival, 06/02/2016 (see Ecarnaçao and Calado 2018); MHNUSC-Bry 437: Algarve: Ferragudo, Portimão, 37.11667°N 08.53333°W, 27/03/2004 (Fig. 2d).

### Description

Colony encrusting, multiserial, unilamellar, or multilamellar; color in life variable, from orange to brownish-purple. Zooids subrectangular, about twice as long as wide, 0.83 to 1.10 mm long by 0.32 to 0.47 mm wide, separated by slightly raised lateral walls. Frontal shield slightly convex, obscured by the cuticle, with numerous round pseudopores; two intrazooidal septula proximolateral to the orifice, each with 3–6 small pores. Orifice large, subcircular to oval, wider than long, 0.21 to 0.27 mm long by 0.25 to 0.29 mm wide, with a well-defined U-shaped sinus; orificial rim thin, sometimes slightly raised; condyles narrow, frequently inconspicuous. Operculum with a broad, biconcave proximal band. Spines, avicularia, and ooecia absent.

### Remarks

In Iberian waters, there are several previous records of *W. subovoidea* and *W. subtorquata*, both from Mediterranean and Atlantic coastlines. The material examined by us, however, coming from localities all along the Iberian Atlantic coast, fits the description of *W. subatra* made by Vieira et al. (2014).

Taking into account the material we examined and the previous references that can be confirmed, *W. subatra* has been recorded from the Bay of Biscay from Santander and Gijon in the intertidal, on pontoons, ropes, and buoys (present work: see material examined and Fig. 2a); in many localities along the Galician coast from the intertidal to 12 m depth (Reverter-Gil and Fernández-Pulpeiro 2001, as *W. subovoidea*; and present work: see material examined and Figs. 2b, c and 3a–c); and in several localities along the southern half of Portugal from the intertidal to 32 m depth (Souto et al. 2014 and Reverter-Gil et al. 2014, both as *W. subtorquata*; and present work: see material examined and Fig. 2d).

There is a nominal record from Isla Grosa (Murcia, Mediterranean) made by Calvín Calvo (1986) as *W. subovoidea*, but the lack of a description or material prevents providing an updated identification. Templado et al. (2002) also reported *W. subovoidea* from the Columbretes Islands (Mediterranean Spain), but the reference material deposited at the MNCN is actually *W. cucullata* (see above). The records from the same archipelago made by d’Hondt (1979) and Saguar and Boronat (1987) as *W. subovoidea* probably belong to the same species. Zabala (1986) reported *W. subovoidea* from the Catalan coast but this record most likely also corresponds to *W. cucullata* (see above). However, we have no further information about the record of *W. subovoidea* from Cabrera (Balearic Islands, 20–52 m depth) made by Zabala (1993), but other material from this archipelago also corresponds to *W. cucullata* (see below).

In summary, *W. subatra* is distributed in the Iberian Peninsula only along the Atlantic coast, from the Cantabrian Sea to the Algarve (south Portugal), from the intertidal down to 32 m depth (Fig. 7). These records represent a continuation towards the south of the distribution noted by Vieira et al. (2014) for *W. subatra* in the NE Atlantic: Helgoland, Ireland, British Channel, and western France.

### *Watersipora arcuata* Banta, 1969b

(Figs. 3d, e, 7)*Watersipora arcuata* Banta, 1969b: 99, figs. 1–7.

*Watersipora subovoidea*: López de la Cuadra 1991: 197 (part, only material from Caños de Meca).

Not *Watersipora subovoidea*: López de la Cuadra 1991: 197 (part, material from El Portil), pl. 2, fig. B, pl. 21, fig. H, pl. 22 fig. A [=*Watersipora souleorum* Vieira et al., 2014].

Not *Watersipora subovoidea*: Gautier 1962: 183 (part: Castiglione, Algeria); Zabala 1986: 396, pl. 6A; Hayward and McKinney 2002: 63, fig. 29A-B; Templado et al. 2002: 203; Chimenz Gusso et al. 2014: 308, fig. 173a-d [=*Watersipora cucullata* (Busk, 1854)].

Not *Watersipora subovoidea*: César-Aldariz et al. 1997: 215, figs. 6 and 7; Reverter-Gil and Fernández-Pulpeiro 2001: 121. [=*Watersipora subatra* (Ortmann, 1890)].

*Watersipora arcuata*: Ferrario et al. 2015: 911, figs. 3 and 4.

### Material examined

**Atlantic, SW Spain**—MHNUSC-Bry 470, 471: Caños de Meca (Cádiz), 36.18494°N 06.01361°W, intertidal, May 1990. López-Fé Coll. (Fig. 3d, e).

### Description

Colony encrusting, multiserial, cream colored after fixation. Zooids subrectangular to hexagonal, 0.74 to 0.96 mm long by 0.27 to 0.43 mm wide, separated by slightly raised grooves. Frontal shield uniformly perforated by round, depressed pseudopores; two intrazooidal septula proximolateral to the orifice, each with 1–3 small pores. Orifice large, semicircular, slightly wider than long, 0.18 to 0.20 mm long by 0.18 to 0.23 mm wide, with proximal margin convex, curved inwards; orificial rim slightly raised distally and laterally; condyles pyramidal in shape, conspicuous, sometimes slightly curved at the tip. Operculum semicircular, dark, with a pair of opercular lucidae (transparent spots). Spines, avicularia, and ooecia absent.

### Remarks

*Watersipora arcuata* was described by Banta (1969b) from California, but the same author (Banta 1969c) considered it as non-indigenous species (NIS), probably introduced since 1963. The potential native origin of *W. arcuata* is thought to be the tropical Eastern Pacific, but nowadays, the species has been reported from Australia, New Zealand, the Pacific coasts of Mexico, California, Hawaii, and the Mediterranean (Ferrario et al. 2015). The presumably first record of the species in European waters was made by Ferrario et al. (2015) based on material collected in 2013 and 2014 in the Ligurian Sea (Italian NW Mediterranean). Shortly after, Ulman et al. (2017) reported *W. arcuata* in, among other Mediterranean localities, the Spanish Mediterranean coast presumably for the first time, based on material collected in marinas at Alicante and Barcelona during November 2016.

We ourselves have revised a sample collected in May 1990 at Caños de Meca (Cádiz, Atlantic side of the Gibraltar Strait area) and reported by López de la Cuadra (1991) in his PhD as *W. subovoidea*. This material corresponds to *W. arcuata* (see Fig. 3d), as already noted by the author himself when he kindly sent us the sample. Accordingly, the introduction of this species in European waters actually occurred at least 25 years before its supposed first European record by Ferrario et al. (2015).

### *Watersipora souleorum* Vieira, Spencer Jones, & Taylor, 2014

**Fig. 4:**
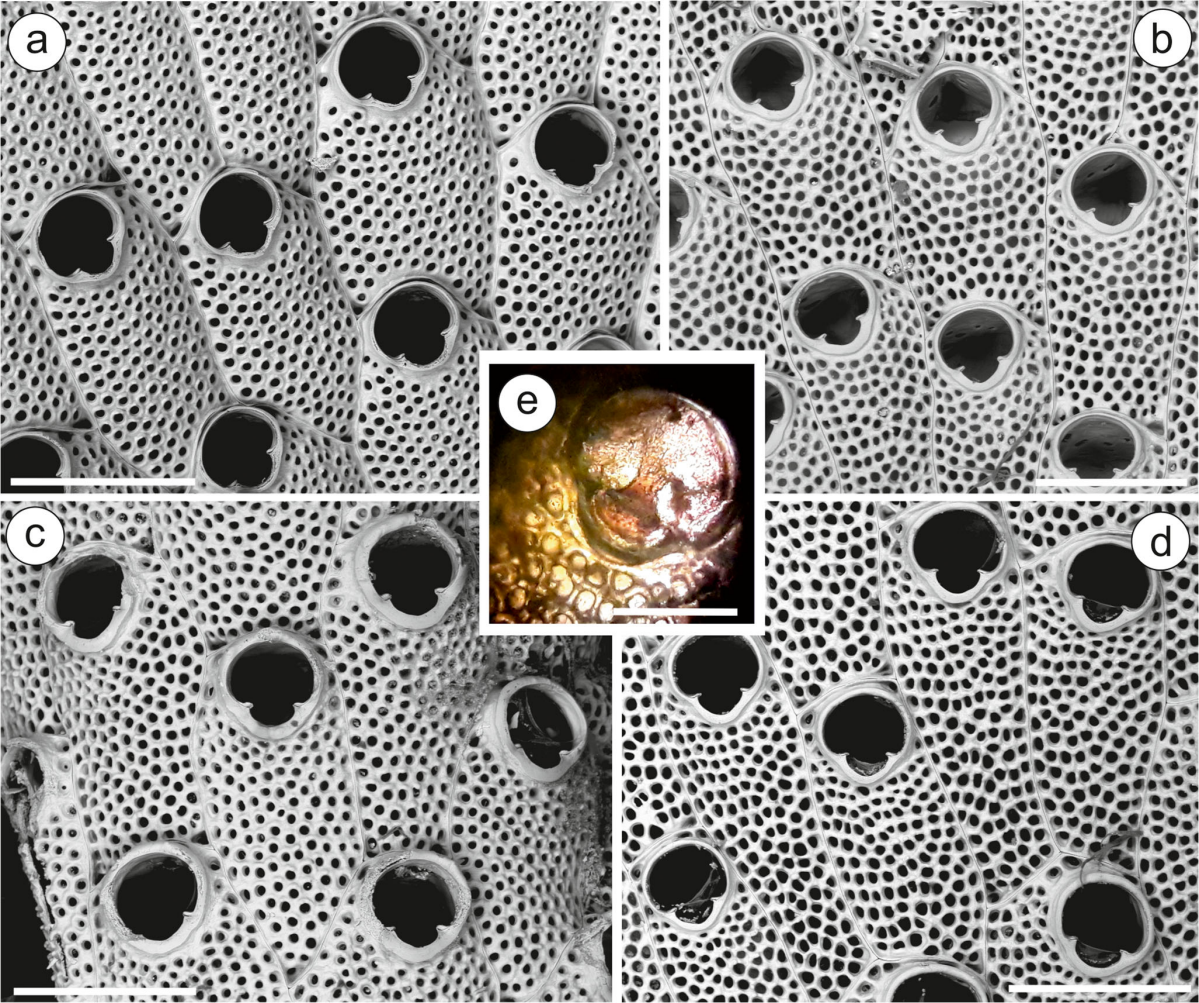
*Watersipora souleorum*. **a** Colonies from Faro (MHNUSC-Bry 438), **b** from Huelva (MHNUSC-Bry 472), **c** from Gibraltar (MNHN-IB-2008-8123), **d** from Marseille (MNHN-IB-2008-11527); **e** detail of the operculum (MNHN-IB-2008-11527: Marseille). Scale bars: **a**, **b**, **c**, **d** 0.5 mm; **e** 0.2 mm

(Figs. 4, 7)* Watersipora souleorum* (Figs. 4, 7) Vieira et al., 2014: 174, figs. 17, 59–64, 68, 71.

*Watersipora cucullata*: Calvet 1931: 113 (part: Gibraltar).

*Watersipora subovoidea*: Gautier 1962: 183 (part: Marseille and Sicilia); López de la Cuadra 1991: 197 (part, material from el Portil), pl. 2, fig. B, pl. 21, fig. H, pl. 22 fig. A.

Not *Watersipora subovoidea*: Gautier 1962: 183 (part: Castiglione, Algeria); Zabala 1986: 396, pl. 6A; Hayward and McKinney 2002: 63, fig. 29A-B; Templado et al. 2002: 203; Chimenz Gusso et al. 2014: 308, fig. 173a-d [=*Watersipora cucullata* (Busk, 1854)].

Not *Watersipora subovoidea*: López de la Cuadra 1991: 197 (part, material from Caños de Meca) [=*Watersipora arcuata* Banta, 1969b].

Not *Watersipora subovoidea*: César-Aldariz et al. 1997: 215, figs. 6 and 7; Reverter-Gil and Fernández-Pulpeiro 2001: 121. [=*Watersipora subatra* (Ortmann, 1890)].

*Watersipora subtorquata*: Souto et al. 2014: 145, fig. 6F (part: Faro); Reverter-Gil et al. 2014: 26 (part: Faro).

Not *Watersipora subtorquata*: Ryland et al. 2009: 55, figs. 3, 4A, C, E, F; Souto et al. 2014: 145 (part: Cascais and Portimão); Reverter-Gil et al. 2014: 26 (part: Cascais and Portimão). [=*Watersipora subatra* Ortmann, 1890].

### Material examined

**Atlantic, SW Iberian Peninsula**—MHNUSC-Bry 438: Laguna of Faro (S Portugal), 37.01139°N 07.99833°W, intertidal, Mars 2004 (Fig. 4a). MHNUSC-Bry 472, 473: El Portil (Huelva), 37.17778°N 07.13055°W, intertidal, Mars 1989. López-Fé Coll. (Fig. 4b). **Strait of Gibraltar**—MNHN-IB-2008-8123: Military port of Gibraltar; *Princesse Alice*, campagne 1894, st. 451, 10 m. Calvet Coll. (as *Watersipora cucullata*) (Fig. 4c). **W Mediterranean**—MNHN-IB-2008-11526: Catania, Sicilia. Gautier Coll. (labeled as *Watersipora cucullata*). MNHN-IB-2008-11527: Old Harbor of Marseille. Gautier Coll. (labeled as *Watersipora cucullata*) (Fig. 4d, e).

### Description

Colony encrusting, multiserial, blackish in color when dry. Zooids subrectangular, about twice as long as wide, 0.74 to 1.00 mm long by 0.28 to 0.57 mm wide, separated by slightly raised grooves. Polypide red (López de la Cuadra 1991, pl. 2, fig. B). Frontal shield slightly convex, obscured by dark cuticle, with numerous, round pseudopores; latero-oral intrazooidal septula absent. Orifice large, oval, slightly wider than long, 0.21 to 0.24 mm long by 0.22 to 0.27 mm wide, with a well-defined U-shaped sinus demarcated by triangular projections; orificial rim thickened around whole orifice; bar-shaped condyles. Operculum with a narrow, biconcave dark central band. Spines, avicularia, and ooecia absent.

### Remarks

Vieira et al. (2014) described the species *W. souleorum* and reported it from several localities in the Atlantic Ocean (Azores, Cape Verde and Senegal), Mediterranean (Naples), and Indian Ocean (India, Sry Lanka and Seychelles). They also included a sample labeled “west coast of Spain, S. Kent coll.” (NHMUK 1872.2.3.147), so this species would be presumably also present in Iberian Atlantic waters. Nonetheless, the labels of the material studied by Saville-Kent are very vague, without indicating precise localities, so the origin of the sample is uncertain.

We have revised the original, small sample of the record made by Calvet (1931) of *W. cucullata* from Gibraltar (MNHN-IB-2008-8123), consisting of about 20 zooids. This colony is here identified as *W. souleorum* (Fig. 4c).

In his PhD, López de la Cuadra (1991) reported material of *W. subovoidea* from SW Spain, now housed at the MHNUSC. Material from el Portil (Huelva) is here identified as *W. souleorum* (Fig. 4b), while material from Caños de Meca (Cádiz) is *W. arcuata* (see above and Fig. 3d). Finally, Souto et al. (2014) and Reverter-Gil et al. (2014) reported *W. subtorquata* from several Portuguese localities. Material from Faro (S Portugal) corresponds to *W. souleorum* (Fig. 4a), while material from Cascais and Portimão is *W. subatra* (see above and Fig. 2d).

According to Vieira et al. (2014), *W. souleorum* seems to have been misidentified as *W. cucullata* several times. For instance, besides the report made under this name by Calvet (1931) from Gibraltar, two samples of the Gautier collection (see material examined), reported by this author in 1962 as *W. subovoidea* (although identified in the labels as *W. cucullata*) are here corrected to *W. souleorum* (Fig. 4d, e). Gautier (1962) even used the material from the Old Harbor of Marseille (MNHN-IB-2008-11527) to give the biometrics of *W. subovoidea* (see Gautier 1962: 183).

### Genus *Terwasipora* gen. nov.

http://zoobank.org/B43B8498-FCC6-45C4-8A88-73D31F41CEAF

### Diagnosis

Colony encrusting, whitish, multiserial, unilaminar to multilaminar. Autozooids subrectangular to hexagonal, flat, separated by narrow sutures. Cryptocystidean frontal shield with numerous rounded pseudopores, surrounded by a strip of gymnocyst. Latero-oral intrazooidal septula proximo-lateral to orifice. Orifice campanulate, without sinus, surrounded by gymnocyst; condyles present. Operculum pale brown, with a distal sclerite and lateral lucidae. Multiporous mural pore plates in distolateral and transverse distal walls. Spines, avicularia, and ooecia absent. Ancestrula with frontal wall completely membranous.

Type species: *Lepralia complanata* Norman, 1864.

Etymology: An anagram of *Watersipora*, the genus in which *Lepralia complanata* was classified for more than 40 years.

### *Terwasipora complanata* (Norman, 1864) comb. nov.

**Fig. 5:**
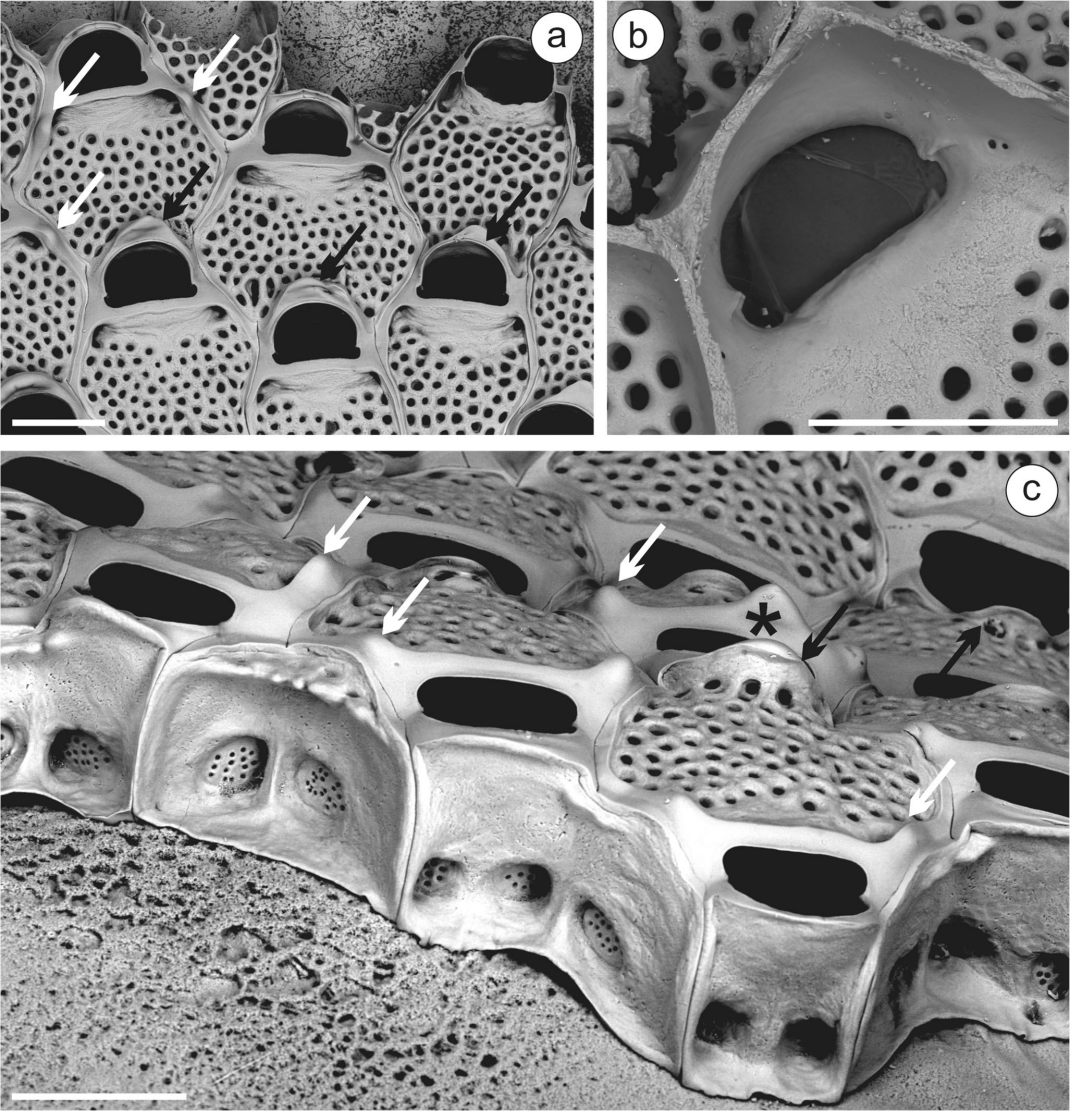
*Terwasipora complanata* comb. nov. (MHNUSC-Bry 616: Cíes Islands). **a** Growing edge of a colony showing the development of the intrazooidal septula; note the strips of gymnocyst surrounding the zooid and the whole orifice, and the early development of the pair of knob-like umbones (white arrows) and of the mid-distal triangular plateau (black arrows); **b** inner view of the primary orifice showing the condyles and intrazooidal septula; **c** growing edge of colony showing the multiporous mural pore plates; note the strips of gymnocyst surrounding the zooid and the whole orifice, and the early development of the pair of knob-like umbones (white arrows), single medio-proximal triangular umbo (asterisk) and of the mid-distal triangular plateau (black arrows). Scale bars = 0.2 mm

**Fig. 6:**
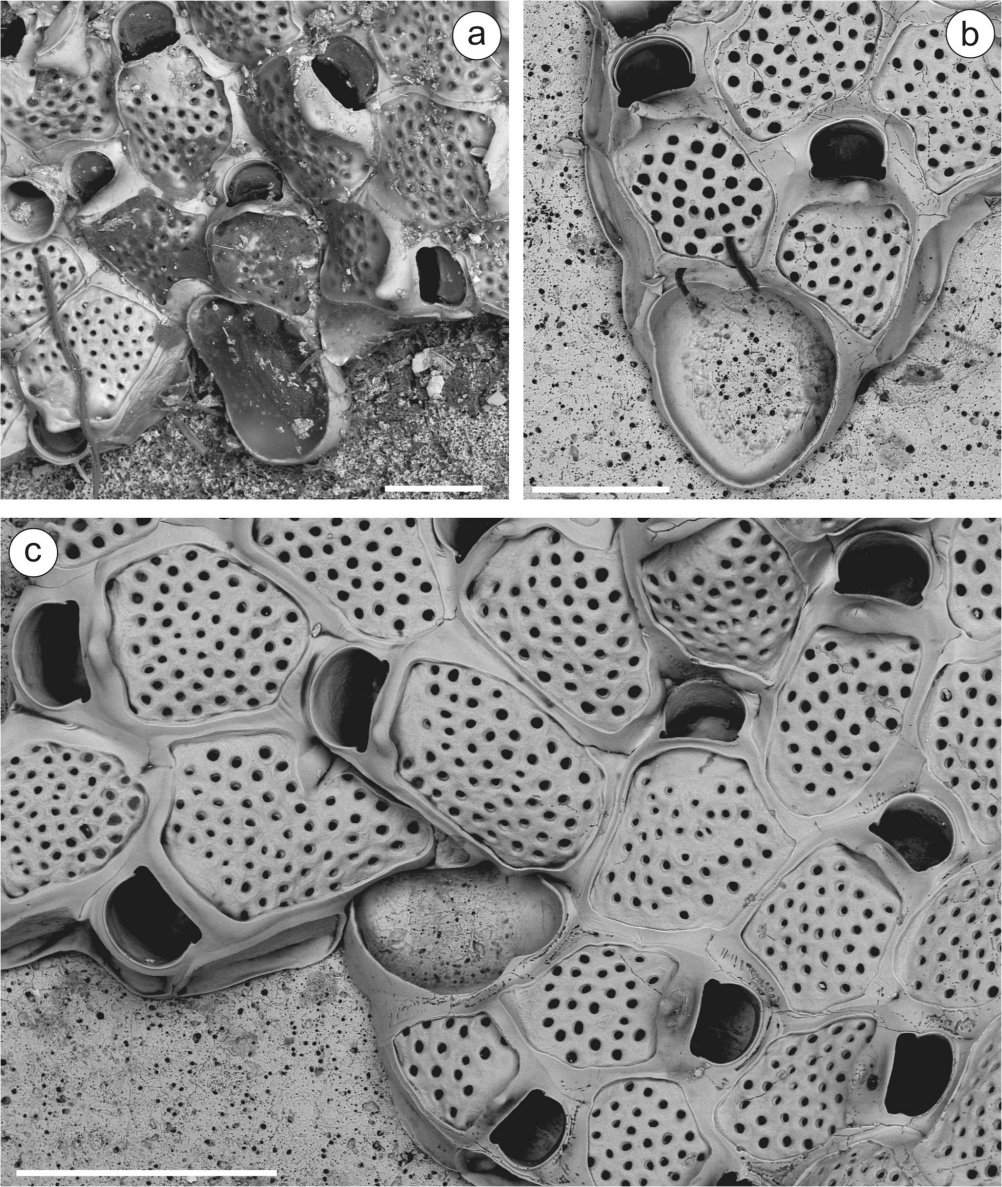
*Terwasipora complanata* comb. nov. (Cíes Islands). **a** Uncleaned colony showing the ancestrula and periancestrular zooids; note the polypide in the ancestrula and the early development of the single medio-proximal triangular umbo (MHNUSC-Bry 615); **b** cleaned ancestrula and periancestrular zooids (MHNUSC-Bry 616); **c** astogenesis; note the strips of gymnocyst surrounding the zooid and the whole orifice, and the single medio-proximal triangular umbo (MHNUSC-Bry 616). Scale bars: **a**, **b** 0.2 mm; **c** 0.4 mm

(Figs. 5, 6 and 8)*Lepralia complanata* Norman, 1864: 85, pl. 10, fig. 4.

*Membranipora smittii* Manzoni, 1870: 333, pl. 3, fig. 16.

*Micropora complanata* (Norman): Hincks 1880: 175, pl. 23, figs. 8 and 9.

*Hippoporina complanata* (Norman): Neviani 1900: 183.

*Cryptosala* (sic) *complanata* (Norman): Canu and Bassler 1925: 34, pl. 7, fig. 12 (*Cryptosula* in the plate).

“*Micropora*” *complanata* (Norman): Gautier 1962: 67.

*Watersipora complanata* (Norman): Hayward 1976: 323, figs. 2 and 3; Zabala 1986: 395, fig. 128; Hayward and Ryland 1999: 192, figs. 74B, 75; Reverter-Gil and Fernández-Pulpeiro 2001: 122; De Blauwe 2009: 372, figs. 397, 398; Chimenz Gusso et al. 2014: 307, fig. 172a-d; Reverter-Gil et al. 2014: 26.

Not *Watersipora complanata* (Norman): d’Hondt 1988: 515.

Not *Watersipora complanata* (Norman): Harmelin and d'Hondt 1992: 29 (part or whole).

### Material examined

**NW Iberian Peninsula**—MHNUSC-Bry 119a, 127, 164: Ría de Ferrol, D12, 43.45889°N 08.29333°W, 20 m depth; MHNUSC-Bry 135b: Ría de Ferrol, D19, 43.46389°N 08.26333°W, 8 m depth; MHNUSC-Bry 150a: Ría de Ferrol, D10, 43.45500°N 08.30889°W, 12 m depth; MHNUSC-Bry 151, 456g: Ría de Ferrol, D13, 43.45583°N 08.29889°W, 9 m depth; MHNUSC-Bry 315: Ría de Ferrol, San Felipe, 43.46694°N 08.27611°W, 8 m depth; MHNUSC-Bry 592: Ría de Ferrol, 43.45889°N 08.30278°W, 18 m depth; MHNUSC-Bry 647, 656, 658: Ría de Ferrol, punta Redonda, 43.46389°N 08.26333°W, 8 m depth; MHNUSC-Bry 5a: Ría de Vigo, V41, 42.23139°N 08.76389°W, 9 m depth; MHNUSC-Bry 24b, 47c, 104b: Ría de Vigo, V2, 42.22944°N 08.88056°W, 23 m depth; MHNUSC-Bry 45a: Ría de Vigo, V3, 42.20833°N 08.89639°W, 8 m depth; MHNUSC-Bry 104a, 465: Ría de Vigo, V29, 42.17083°N, 08.82889°W, 19 m depth; MHNUSC-Bry 104c: Ría de Vigo, V19, 42.25139°N 08.89528°W, 12 m depth; MHNUSC-Bry 128e: Ría de Vigo, no more data; MHNUSC-Bry 597, 615 (Fig. 6a): Ría de Vigo, Islas Cíes, 42.21436°N 08.90442°W, 8 m depth; MHNUSC-Bry 599, 606, 609, 614, 616–620: Ría de Vigo, Islas Cíes, 42.21150°N 08.90567°W, 12 m depth (Figs. 5 and 6b, c); MHNUSC-Bry 603, 607, 611: Ría de Vigo, Islas Cíes, 42.20450°N 08.90696°W, 5 m depth; MHNUSC-Bry 604, 610, 613: Ría de Vigo, Islas Cíes, 42.22883°N 08.89511°W, 6 m depth; MHNUSC-Bry 622: Ría de Vigo, Islas Cíes, 42.43871°N 08.89948°W, 6 m depth; MHNUSC-Bry 628: Ría de Vigo, Islas Cíes, 42.23523°N 08.89827°W, 5 m depth. **Portugal**—(MHNUSC, material without reference number): Berlengas, St. 23, 39.42065°N 09.53478°W, 21 m depth (M12 B ST 23 EB 05); Berlengas, St. 24. 39.42153°N 09.53448°W, 20 m depth (M12 B ST 24 EB 35); Berlengas, St. 27, 39.41887°N 09.50217°W, 15 m depth (M12 B ST 27 B 88); Berlengas, St. 28, 39.41963°N 09.49870°W, 25 m depth (M12 B ST 28 B 17); Berlengas, St. 29, 39.41910°N 09.50575°W, 14 m depth (M12 B ST 29 B 22); Berlengas, St. 31, 39.42072°N 09.50883°W, 19 m depth (M12 B ST 31 B 35); Berlengas, St. 37, 39.41148°N 09.50667°W, 21 m depth (M12 B ST 37 B 28); Berlengas, St. 48, 39.42438°N 09.52500°W, 21 m depth (M12 B ST 48 B 17); Arrabida, St. 12, 38.47778°N 08.97235°W, 6 m depth (ST12bry23); Arrabida, St. 34, 38.42970°N 09.10270°W, 30 m depth (ST34bry14); JS, personal collection: Cascais, St. 39, 38.67810°N 09.34647°W, 6.7 m depth (B039 02); Cascais, St. 10, 38.69167°N 09.37167°W, 5 m depth (B01013). **NW Mediterranean**—MNHN-IB-2008-10840, 10841: Banyuls-sur-Mer, Gautier. Coll.; MNHN-IB-2008-10839: Marseille, Gautier. Coll.; MNHN-IB-2008-10838: Corse, Gautier. Coll.

*Other material examined*: MNHN-IB-2008-13276: Balgim DR115, *Steraechmella buski*, d’Hondt Coll.; MNHN-IB-2008-13588: Balgim DW50, *Steraechmella buski*, *Herentia thalassae*, d’Hondt Coll.; MNHN-IB-2008-15384: Estepona, 200 m, several species, d’Hondt Coll.

### Description

Colony encrusting, unilaminar to multilaminar by overgrowing, forming broad, irregular, whitish to cream-colored incrustations, mainly on shells and rocks.

Autozooids sub-rectangular to hexagonal or rhomboidal, 0.60 to 0.85 mm long by 0.35 to 0.60 mm wide, in alternating series separated by narrow sutures (Figs. 5a and 6c). Frontal shield flat, cryptocystidean, finely granular, closely punctured by small rounded pseudopores, and covered by a thin, transparent cuticle (Fig. 6c). Frontal shield and orifice surrounded by a continuous strip of smooth, gymnocystidean calcification (Figs. 5a, c and 6). Primary orifice campanulate, wider than long, 0.12 to 0.15 mm long by 0.19 to 0.23 mm wide; anter D-shaped, with edge slightly raised; proximal border straight or slightly convex (Figs. 5a, b and 6). Two small latero-proximal condyles (Fig. 5b). Operculum pale brown, with a distal sclerite and two proximal lucidae adjacent to the condyles. Successive gymnocystal calcification of the orifice rim produces a raised, recurved ridge which, in the corners between it and the lateral walls, may overarch to give an impression of shallow opesiular indentations, which actually correspond to latero-oral intrazooidal septula, visible internally (Fig. 6a, b); in later ontogeny, a pair of knob-like umbones proximo-lateral to orifice may appear, or a single, medio-proximal triangular umbo (Figs. 5a, c and 6); and, especially in older zooids, a broadened triangular plateau mid-distally, which has been confused in the past with an ovicell (Fig. 5a). Multiporous mural pore plates in distolateral and transverse distal walls (Fig. 5c). Avicularia and spines absent. Ooecia absent; according to Waters (1925), the embryos are brooded within the maternal zooid. Ancestrula oval, about half the size of autozooids, 0.30 to 0.40 mm long by 0.23 to 0.29 mm wide, with lateral walls calcified, very thin, and frontal surface completely membranous. Distal wall forming an arch coextensive with the operculum. Outline asymmetrical, with the lateral wall of the side of the first bud more curved (Fig. 6a, b). Ancestrula budding one lateral and one distolateral daughter zooids; subsequent astogeny asymmetrical, spiraling to one side or another, with zooids increasingly larger (Fig. 6c).

### Remarks

*Watersipora complanata* is a relatively well-known species distributed in the Atlantic-Mediterranean region: Adriatic (Novosel and Požar-Domac 2001), western Mediterranean (Gautier 1962; Rosso and Di Martino 2016), Morocco (Canu and Bassler 1925), western Iberian Peninsula (Reverter-Gil and Fernández-Pulpeiro 2001; Reverter-Gil et al. 2014), Brittany (Reverter et al. 1995), and southwest of the British Isles (Hayward and Ryland 1999).

More specifically, in Iberian waters (Fig. 8), it has been recorded with certainty from Galicia, where it is a very common species on shells and stones from the intertidal zone to 56 m depth (Reverter-Gil and Fernández-Pulpeiro 2001, and present work); several localities in Portugal (Reverter-Gil et al. 2014, and present work); in the area of the Strait of Gibraltar (López de la Cuadra and García-Gómez 1988; López de la Cuadra 1991); and in Catalonia (Zabala 1986). It has been also recorded in the southern French Mediterranean coast near Spain, in Banyuls-sur-Mer (Gautier 1962; see material examined).

There are also some records of *W. complanata* from the Ibero-Moroccan Bay at 332–523 m depth (Harmelin and d'Hondt 1992), a depth range that seems excessive for a species that apparently prefers shallow waters. We have revised the only two samples held at the MNHN, one from BALGIM st. DW50 (MNHN-IB-2008-13588) and other from st. DR115 (MNHN-IB-2008-13276), both identified by J.-L. d’Hondt and reported by Harmelin and d’Hondt (1992). The former includes an ovicelled colony of *Steraechmella buski* Lagaaij, 1952 and a colony of *Herentia thalassae* David & Pouyet, 1978. The latter is only a colony of *S. buski*. Taking into account the greater depths of these stations and the misidentifications detected, we consider that the record of *W. complanata* from the Ibero-Moroccan Bay is incorrect. In the same work, the species was also reported from two other stations in the Moroccan Alboran Sea: DR151 and DW132, between 110 and 170 m depth. The first station was also reported by Harmelin et al. (1989). As no material seems to have been preserved, these records cannot be revised. *Watersipora complanata* was also reported by d’Hondt (1988) from a locality near Estepona (Spanish Alboran Sea) at 200 m depth. The reference sample, MNHN-IB-2008-15384, contains many shell fragments with different species, but among them, no colony of *W. complanata* was found. There is only a dead, eroded colony, with orifices full of sand, which at first sight may resemble a *Watersipora*, but presents ovicells and probably represents a smittinid species. Therefore, this record of *W. complanata* by d’Hondt (1988) is here considered incorrect. Finally, the species was also reported by Calvet (1896) in the Bay of Biscay at 180 m depth (‘Caudan’ expedition, St. 17), but taking into account the great depth and the lack of material, this record is here considered doubtful.

Accurate descriptions of *L. complanata* are available in different papers (e.g., Gautier 1962; Fernández Pulpeiro and Rodríguez Babío 1980; Hayward and Ryland 1999; Hayward and McKinney 2002). Until now, however, the ancestrula and the first stages of development were unknown. Moreover, the presence of a strip of gymnocyst surrounding the autozooid had been overlooked by previous authors. In fact, a gymnocyst has never been reported in any *Watersipora* species. These two characters are important to establish the taxonomic placing of this species, which was uncertain for many years. It was described by Norman (1864) as *Lepralia complanata* from an unknown British locality. Shortly after, Manzoni (1870) described a fossil of an extinct species, *Membranipora smittii* that, according to Hincks (1880), is a synonym of *L. complanata*. The later author also transferred the species to the genus *Micropora* Gray, 1848, in which the species would remain for many years. Neviani (1900) proposed to transfer the species to the genus *Hippoporina* Neviani, 1895; Canu and Bassler (1925) to their new genus *Cryptosula* (although it appears erroneously written as *Cryptosala* [sic] *complanata* in Canu and Bassler 1925: 34); and Waters (1925) reported the species again as *Lepralia complanata* from several Mediterranean localities. However, these changes do not seem to have had any impact in the later literature. Gautier (1962) reviewed previous systematic accounts but did not decide upon the affinities of the species, naming it “*Micropora*” *complanata*. Ryland (1969) stated that the species is not a *Micropora* but an ascophoran, and proposed that it could still be called *Lepralia*, though temporarily. Finally, Hayward (1976) transferred it to the genus *Watersipora* based on the nature of the frontal wall, the structure of the orifice and the presence of characteristic communication organs in the lateral and transverse vertical walls. Since then, all the literature refers to the species as *Watersipora complanata*. However, Ryland et al. (2009) already indicated that “Lepralia complanata *Norman* (1864), *currently placed in* Watersipora (*Hayward and Ryland *1999*)*, *with a campanulate orifice, does not appear obviously congeneric with the* arcuata*/*subovoidea*/*subtorquata *group, to which the type species belongs*.”

The situation took a new turn with the recent redescription of the genus *Watersipora* by Vieira et al. (2014). Those authors give a complete and corrected diagnosis of the genus, which in practice leaves out *L. complanata*. Major differences are:

- The ancestrula of *Watersipora* is schizoporelloid, while in *L. complanata* its frontal wall is completely membranous (Fig. 6).
- The orifice in *Watersipora* is subcircular to oval, many times sinuate, while in *L. complanata* it is campanulate, without sinus (Figs. 6a, b and 5b, c).
- In *L. complanata*, there is a strip of smooth gymnocyst surrounding the whole autozooid and the orifice (Figs. 5c and 6). In *Watersipora*, a gymnocyst surrounding the zooid is lacking; only in some species, there is an occasional gymnocystal calcification around the orifice (see, e.g. Vieira et al. 2014, fig. 19).

Other differences are: the operculum of *L. complanata* is pale brown, with a distal sclerite, while in *Watersipora*, it is reddish-brown to black; the colony of *L. complanata* is whitish to cream-colored, not reddish-brown to black as in *Watersipora*. And finally, Vieira et al. (2014) indicated the occasional presence in *Watersipora* of intrazooidal septula at proximal corners of frontal shield, which are absent in *L. complanata*. These differences are sufficient to separate *L. complanata* in a new genus, *Terwasipora* gen. nov., which, however, shares some characters with *Watersipora*. Besides some general features (colony encrusting, multiserial; autozooids flat, sub-rectangular to hexagonal; spines, avicularia and ooecia absent, with embryos brooded internally), both genera share the cryptocystidean frontal shield with numerous rounded pseudopores; the intrazooidal septula proximo-lateral to orifice; the operculum with lucidae; and the multiporous mural pore plates in disto-lateral and transverse distal walls. For these reasons, we tentatively place *Terwasipora* gen. nov. in the family Watersiporidae.

*Terwasipora complanata* comb. nov. also shows some similarities with *Cryptosula pallasiana* (Moll, 1803), type species of the genus *Cryptosula* Canu & Bassler, 1925 (e.g., frontal wall, orifice shape, gymnocyst around the orifice, condyles). However, this species lacks a gymnocyst surrounding the zooid and the intrazooidal septula, the suboral umbo is formed by the cryptocyst, not by the suboral gymnocyst, and presents a schizoporelloid ancestrula. In our opinion, these differences (especially the intrazooidal septula) not only differentiate both genera, but also prevent placing the genus *Terwasipora* in the family Cryptosulidae.

Hayward and Ryland (1999 as *W. complanata*) indicated that the biology and ecology of this species was unknown. We ourselves have not found isolated ancestrulae in the material studied from Galicia and Portugal, but did find small juvenile colonies, composed of the ancestrula and few zooids (Fig. 6), which we observed in late August in the Cíes Islands, and in June and September in the Ría de Ferrol (Reverter Gil 1995 and present work). In the Ría de Ferrol, more than 1300 colonies of *T. complanata* comb. nov. on broken shells were collected, but only 125 on small stones, and only a few on other substrates (Reverter Gil 1995, as *W. complanata*). In the Ría de Vigo, however, the species seems to prefer stones over shells (Barcia Leal et al. 1993, as *W. complanata*), as was also the case in Berlengas Islands (present work). Gautier (1962, as *“M”. complanata*) reported the species from the first 50 m depth in the western Mediterranean, while in Galicia, where this species is very abundant, it was collected from the intertidal zone to 56 m depth (Reverter-Gil and Fernández-Pulpeiro 2001, as *W. complanata*). Thus, *T. complanata* comb. nov. seems to be a shallow-water species that lives on hard substrata near the shore, especially on shells and stones.

## Discussion

**Fig. 7:**
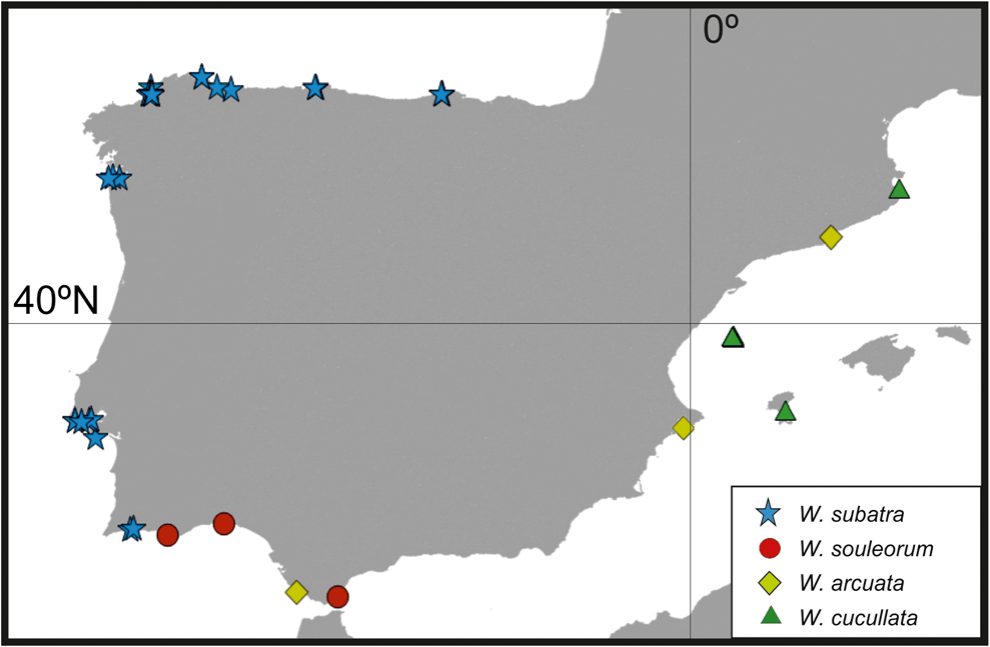
Iberian distribution of *Watersipora* species

Of all the *Watersipora* species found along the Iberian coast, only *W. cucullata* seems to be a native species—it is probably endemic to the Mediterranean (Vieira et al. 2014) (Fig. 7). The other three species of the genus treated here (*W. subatra*, *W. arcuata* and *W. souleorum*) are alien species that have been introduced in different ways and times.

*Watersipora subatra* was originally described from Japan by Ortmann (1890) but the origin of the species is unclear (see Vieira et al. 2014 for details). *Watersipora subatra* is easily dispersed as fouling organism on vessels (Mackie et al. 2012, as *W. subtorquata*) and exhibits a high capacity to grow on artificial substrates with anthropogenic disturbance (Viola et al. 2018). Vieira et al. (2014) considered that *W. subatra* is the most common putatively invasive species of *Watersipora* in Britain, Australia, New Zealand, and California. In the Iberian Peninsula, it seems to be a relatively recent immigrant, perhaps first introduced in the northwest (Fig. 7). In fact, the first Iberian record in 1996 from the coast of Lugo, NW Spain (César-Aldariz et al. 1997 as *W. subovoidea*), was also the first record of *W. subatra* in European waters. We are certain that the species was not present earlier in Galicia because in those years, continuous sampling was carried out on these coasts and a species as characteristic as *W. subatra* would not have gone unnoticed. Moreover, in the same area, two other foreign species appeared at the same time: *Tricellaria inopinata* d’Hondt & Occhipinti Ambrogi, 1985, most likely introduced from the Venetian lagoon together with clams for cultivation (Fernández-Pulpeiro et al. 2002); and *Antarctothoa galaica* (César-Aldariz, Fernández-Pulpeiro and Reverter-Gil, 1999), probably introduced from the Southern Hemisphere via shipping (Hughes et al. 2008). The three species not only survived in Galicia, but they have also dispersed (Reverter-Gil et al. 2019 and present work). *Watersipora subatra* has been slowly extending westward along the Galician coast: Xove in 2007; estuaries of Ferrol and Vigo in 2010; and Cíes Islands in 2012 (see material examined and Fig. 7). Recently, the species has also been collected in Santander in 2018 together with *T. inopinata*; note that in previous samplings in that locality, *W. subatra* was not found. More recently, indeed, the species was also collected in Gijón together with *T. inopinata* and *Beania serrata* Souto, Nascimento, Reverter-Gil & Vieira, 2019. This last species, although described from NW Spain, is considered to be a recently introduced species in the area (Souto et al. 2019). New samplings are required along the Cantabrian coast.

In northern Spain, the species has been collected in seven marinas and harbors, but also in eight natural habitats always located in the internal part of the rías or in areas with a low exposition to waves. The species was never collected in external (exposed) localities of the rías, in spite of the numerous sampling points visited. Also, on the NW Iberian coast, a colony was observed growing on a plastic fragment washed upon a beach (Cabo Prior) (see material examined of *W. subatra*), showing the dispersal capacity of *W. subatra* by rafting on floating objects. Anyway, in view of the records along the northern coast of Spain, we think that the fouling on hulls of leisure and commercial ships is the main path of introduction and dispersion of this species. This conclusion is also supported by the results along the coast of Portugal, where the species does not seem to have been present until recently and had been mainly found in marinas and harbor areas. The only exception occurred in south Portugal, where *W. subatra* was collected growing on the wrecks in the Ocean Revival underwater park (for details of the park and experiments see Encarnaçao and Calado 2018). The first record of *W. subatra* in Portugal was in Portimão (Algarve) in 2004 (Souto et al. 2014, as *W. subtorquata*); it was subsequently recorded in Cascais in 2012 (Canning-Clode et al. 2013, as *W. subtorquata*). Later on, during the campaign Marbis/EMPC 2014 on the Arrábida coast, bryozoans were collected in 17 localities (13 natural habitats, one shipwreck, one directional light signal, one dredged area and only one marina: port of Sesimbra). *Watersipora subatra* was found only in the marina of Sesimbra, growing on pontoons and buoys. This area of the Arrábida had been sampled regularly over many years by Luiz Saldanha, who never recorded the species (Saldanha 2003). In 2015, *W. subatra* was collected again at the coast of Lisbon during the campaign Marbis/EMPC 2016. A total of 64 species of bryozoans were collected at 23 localities. Seventeen of these localities were natural habitats, one was a shipwreck, and five localities were ports and marinas with artificial substrates (concrete, fiberglass pontoons, hulls and metal structures) and natural substrates (mainly rocks and shells). *Watersipora subatra* was detected only in these five marinas or harbor areas (Alcántara, Belén, Cascais, Oeiras, and Almada) growing on all studied substrates (see material examined). On the other hand, recent samplings in natural habitats along the northern half of the Portuguese coast did not detect the species (Souto et al. 2014). The sequence of records does not necessarily show the real sequence of introduction because the species could go unnoticed; nonetheless, previous samples in the same areas did not find this (or other similar) species, so that we can confirm that the introduction is recent. The species may have arrived in Portugal from the north (Galicia), or independently. The differences in the distribution of *W. subatra* between northern Spain, where it appears on artificial and natural habitats (internal areas of rías), and the Portugal coast, where its presence is restricted to marinas and harbors, indicates that other parameters, which have not been taken into account here, may influence the success of the introduction of this species and its extension.

*Watersipora subatra* has not yet been reported from the Mediterranean Sea (Vieira et al. 2014). Based on the currently available data, we can neither confirm nor deny the presence of *W. subatra* in the Iberian Mediterranean. Although there are previous citations of *Watersipora* on that coast, their identity, as stated above, is almost always doubtful. Confirmed records in this area actually correspond to *W. cucullata*.

Previous records of *W. arcuata* on the European coast were made based on material collected in marinas in recent years (Ferrario et al. 2015, 2018; Ulman et al. 2017), proving that it is an introduced species in European waters. The native origin of this species is thought to be the tropical Eastern Pacific, but nowadays, the species has been reported worldwide (Ferrario et al. 2015). The material cited here was collected in 1990, near Trafalgar, although it was reported by López de la Cuadra (1991) as *W. subovoidea*. Interestingly, the material was collected on a rocky coast that was not much exposed, with several other bryozoan species, but far from marinas or other harbor facilities. The nearest one is the small port of Barbate, whose main activity is inshore fishing. The locality is, however, near the Gibraltar Strait (see Fig. 7), an area with major maritime traffic, which is assumed to be one of the most important vectors of unintentional NIS introduction worldwide.

There are few previous records of *W. souleorum* worldwide (Vieira et al. 2014). There are two old samples from Iberian waters. One sample, revised by Vieira et al. (2014), was collected by Saville-Kent around 150 years ago. As the label only states “west coast of Spain”, its origin is uncertain, being in the NW (i.e. Galicia) or perhaps in the SW (i.e. west Andalusia), the latter being more probable in view of other Iberian records, as explained below. The other sample, reported here, was collected in 1894 in Gibraltar (MNHN-IB-2008-8123), although it was reported later by Calvet (1931 as *W. cucullata*) (Figs. 4c and 7). The fact that it was collected at the military port points to an introduced species. The other two Iberian records of the species come from very different localities (Fig. 7). One stems from the intertidal zone at the Laguna of Faro (S Portugal), an area with a high anthropogenic disturbance. This material was collected in 2004 and reported 10 years later (Souto et al. 2014 as *W. subtorquata*). The other sample comes from El Portil (SW Spain), collected in 1989 on a rocky shore near the mouth of a river, but unaffected by significant human activity. This material was reported by López de la Cuadra (1991) as *W. subovoidea*. Although the distribution of *W. souleorum* probably implies an artificial dispersion, nothing is known about the origin of the species (Vieira et al. 2014).

The Gulf of Cádiz turns out to have a great diversity of *Watersipora* species considered as NIS: *W. subatra* in Portimão, *W. souleorum* in Faro, Huelva and Gibraltar and *W. arcuata* in Trafalgar (Fig. 7). Even *W. cucullata* was reported in Gibraltar, by Barroso (1917), although this is a native species and the record has not been checked. As some of these records are old, previous to the recent expansion of *W. subatra* in Iberian waters, it would be necessary to take new samples in these or other localities to determine the current situation.

**Fig. 8:**
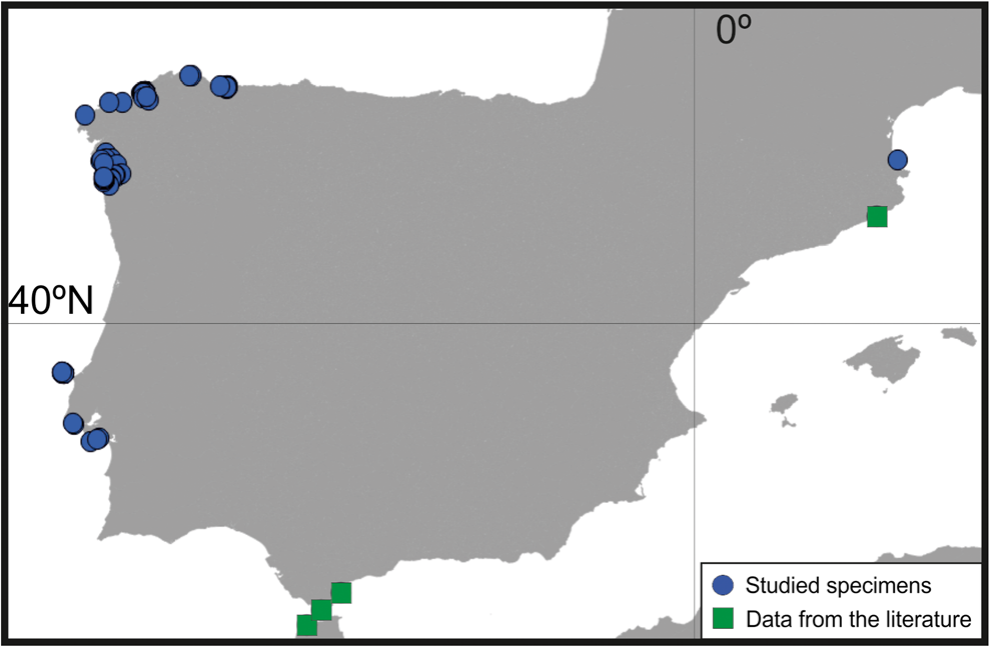
Iberian distribution of *T. complanata* comb. nov

The last species treated here, *T. complanata* comb. nov., was first described in Britain and was recorded in the Atlantic-Mediterranean region, so it must be considered as a native species in Iberian waters (Fig. 8).

## Conclusions

At present, four species of *Watersipora* are known to exist in Iberian waters (Fig. 7). *Watersipora cucullata* is the only native Iberian species, present on the Spanish Mediterranean coast in Catalonia, Valencia, and the Balearics between 20 and 42 m depth, but it seems to be absent from the Atlantic. There are several old records of *Watersipora* in the Iberian Mediterranean coast that perhaps also correspond to this species, but it would be necessary to collect new material from the Iberian Mediterranean coast to shed light on its presence in areas where it has been reported in the past or in new sites. In contrast, *W. subatra* seems to have been introduced in Iberian and European Atlantic waters relatively recently (ca. 1996) and have been expanding to a large part of the Iberian Atlantic coast, but also later to other European Atlantic localities. This species is apparently an alien species absent from the Mediterranean, but new samplings are required here, as well as both along the north coast (Cantabrian Sea) and in the southwest (Gulf of Cádiz) to determine whether the species is still expanding in Iberian waters. A third species, *W. arcuata*, was collected for the first time in Europe at Huelva (Spanish Atlantic coast) in 1990 and recently in Mediterranean marinas. New sampling is needed to determine if the species is still present in the Iberian Atlantic coast 30 years later, and its probable extension to other harbor facilities along the Iberian Mediterranean coast. Finally, a fourth species, *W. souleorum*, is known in Iberian waters from only two localities in the Gulf of Cadiz, and one in the Gibraltar Strait, but collected 125 years ago. Again, new sampling is needed to determine its current distribution.

The recent redescription of the genus *Watersipora* excludes *L. complanata*, and therefore we define a new watersiporid genus for the species, *Terwasipora* gen. nov. Although the species is considered widely distributed in the western Mediterranean (Gautier 1962), in Iberian waters *T. complanata* comb. nov. is much more abundant along the Atlantic coast, especially in the northwest (Galicia). In contrast, along the Iberian Mediterranean, there is only a single record of the species from Catalonia along with one from a very nearby French locality (Banyuls-sur-Mer) (Fig. 8). In our opinion, however, this taxon can still be considered as an Iberian native species. *Terwasipora complanata* comb. nov. seems to be a shallow-water species inhabiting hard substrata near the shore, especially on shells and stones, down to ca. 50 m depth.
